# Human collecting lymphatic glycocalyx identification by electron microscopy and immunohistochemistry

**DOI:** 10.1038/s41598-023-30043-x

**Published:** 2023-02-21

**Authors:** S. Gianesini, E. Rimondi, J. D. Raffetto, E. Melloni, A. Pellati, E. Menegatti, G. P. Avruscio, F. Bassetto, A. L. Costa, S. Rockson

**Affiliations:** 1 Department of Translational Medicine, LTTA Centre, University of Ferrara, Ferrara, Italy; 2 Department of Surgery, Uniformed Services University of Health Sciences, Bethesda, USA; 3 Surgery Department, VA Boston Healthcare System, Harvard University, Boston, USA; 4 Environmental Sciences and Prevention Department, University of Ferrara, Ferrara, Italy; 5 Department of Cardiac, Thoracic and Vascular Sciences, Hospital-University of Padua, Padua, Italy; 6 Department of Neuroscience, Clinic of Plastic Surgery, University of Padova, Padua, Italy; 7 Division of Cardiovascular Medicine, Stanford University School of Medicine, Stanford, USA

**Keywords:** Translational research, Electron microscopy

## Abstract

Blood flow is translated into biochemical inflammatory or anti-inflammatory signals based onshear stress type, by means of sensitive endothelial receptors. Recognition of the phenomenon is of paramount importance for enhanced insights into the pathophysiological processes of vascular remodeling. The endothelial glycocalyx is a pericellular matrix, identified in both arteries and veins, acting collectively as a sensor responsive to blood flow changes. Venous and lymphatic physiology is interconnected; however, to our knowledge, a lymphatic glycocalyx structure has never been identified in humans. The objective of this investigation is to identify glycocalyx structures from ex vivo lymphatic human samples. Lower limb vein and lymphatic vessels were harvested. The samples were analyzed by transmission electron microscopy. The specimens were also examined by immunohistochemistry. Transmission electron microscopy identified a glycocalyx structure in human venous and lymphatic samples. Immunohistochemistry for podoplanin, glypican-1, mucin-2, agrin and brevican characterized lymphatic and venous glycocalyx-like structures. To our knowledge, the present work reports the first identification of a glycocalyx-like structure in human lymphatic tissue. The vasculoprotective action of the glycocalyx could become an investigational target in the lymphatic system as well, with clinical implications for the many patients affected by lymphatic disorders.

## Introduction

The vascular endothelium has been defined as an organ, reflecting its multiple influences upon vessel contraction, cellular and nutrient trafficking, coagulation balance and angiogenesis^1^. These phenomena are associated with the interaction between the flow force and the related endothelial release of biochemical products. The translation of shear stress into various endothelial phenotype expressions is known as mechanochemical transduction^2^. While laminar flow leads to anti-inflammatory endothelial expression, turbulent flow generates pro-inflammatory, pro-thrombotic and pro-atherosclerotic signaling^3^. The glycocalyx (GCX) is a multi-component structure known to cover the endothelial surface of the blood vasculature, acting as an extremely sensitive receptor to flow dynamics^4^.

The GCX has been recognized as a fundamental component of the arterial and venous endothelium, which is involved in various pathophysiologic processes and acknowledged as a potential therapeutic target for multiple diseases^5^. It can be visualized as a carbohydrate-rich layer anchored to the endothelium with proteins that protect and interact with the vessel wall. Proteoglycans and glycoproteins are thus anchored to the endothelium, around which soluble components from both the plasma and the same vessel wall interact in a dynamic balance with the GCX. Proteoglycans are usually comprised of a core protein attached to glycosaminoglycan chains. Syndecans and glypicans act as core proteins. There are also glycoproteins with acidic oligosaccharides and terminal sialic acid conjugates. These are represented among the endothelial bound proteins with a polypeptide backbone that form various adhesion molecules, selectins, and integrins^4–6^. The glycosaminoglycan chains vary according to several anatomical and hemodynamic conditions. There are five sulfated glycosaminoglycan chains: heparan sulfate, dermatan sulfate, chondroitin sulfate, keratan sulfate, and heparin, and one non-sulfated hyaluronic acid. Heparan sulfate proteoglycans represent approximately 50–90% of the total GCX proteoglycan content, and in humans most endothelial cell glycosaminoglycans are heparan sulfate, chondroitin sulfate, and hyaluronic acid^7,8^. The second most common is chondroitin sulfate/dermatan sulfate, usually reported in a 1: 4 ratio when compared to heparan sulfate^9^. Additionally, the glycoproteins anchor the GCX to the endothelium and these glycoproteins include cell adhesion molecules (integrins and selectins) as well as coagulation components. The thickness, structure, and electronic charge of the GCX regulates the permeability of the endothelium^10^.

Being negatively charged, the GCX repels red blood cells and platelets, limiting contact with the endothelium and favoring a laminar flow^11,12^. The GCX has also been demonstrated to regulate leukocyte adhesion to the endothelium^13^. The several components of the GCX regulate phenotypic expression of endothelial microenvironment phenotype, including the balance of the lipolytic^14^ and anticoagulation systems^15^. Moreover, the GCX expresses a vasculo-protective role, due to its ability to modulate inflammatory responses by regulating cytokine binding^16^ and as a quencher of oxygen radicals^17^. Studies evaluating the molecular functions of the GCX and its involvement in the pathophysiology of several arterial (diabetes, ischemia, atherosclerosis) and venous (chronic venous insufficiency, venous thrombosis) diseases has been demonstrated^18–24^.

Changes in the flow characteristics lead to alterations in the glycocalyx signaling, which is involved with endothelial protection and permeability regulation, leukocyte adhesion and diapedesis, vessel contraction and coagulation balance modifications (platelet adherence inhibition, coagulation activation)^25^. At a systemic level, the GCX also has demonstrable immune and tissue healing functions^6,26^. GCX alteration also leads to dysregulation of permeability and coagulation balance, together with enhanced leukocyte adhesion, which are all phenomena involved in venous disease pathophysiology^27,28^.

The capillary exchange process once comprehended chiefly through the Starling principle is now known to include an active process modified by the GCX as a sieve of various porosities, thereby regulating the vessel permeability to macromolecules and implicated in eventual edema formation^29,30^. The selective semipermeable GCX membrane in the venous post capillary venule causes the reabsorption of the majority of the interstitial fluid through the lymphatic system^31^. This latter observation led our research group to search for previous studies that attempted to detect the presence of the GCX in the lymphatic system.

Through an extensive search dating back to the early 1960s, when Rambourg provided evidence that the GCX was present on the surface of cells harvested from rats^18^, the only paper we found on the topic is from an animal model in the murine cremaster muscles and based on confocal microscopic analysis of GCX moieties^32^. We found no publication of an ex vivo human electron microscopic identification and immunohistochemical characterization of the lymphatic GCX, which is the primary aim of this study.

## Materials and methods

A lower limb vein- and lymphatic-containing tissue sample was harvested from a 51 year-old female patient (BMI 25) undergoing a thigh-plasty procedure that was performed following bariatric surgery (sleeve gastrectomy). Apart from medical obesity, no significant comorbidities were reported. The patient was not a smoker. After obtaining informed consent, according to the Declaration of Helsinki on biomedical research involving human subjects, a periprocedural injection of patent blue was performed for in situ anatomic identification of the lymphatic vessels. Ten minutes before the surgical incision, the intradermal and subcutaneous injection of 2.5 ml of patent blue was completed at two different points in the medial region of the thigh. The excess skin was removed in agreement with the preoperative surgical planning.

**Figure 1:**
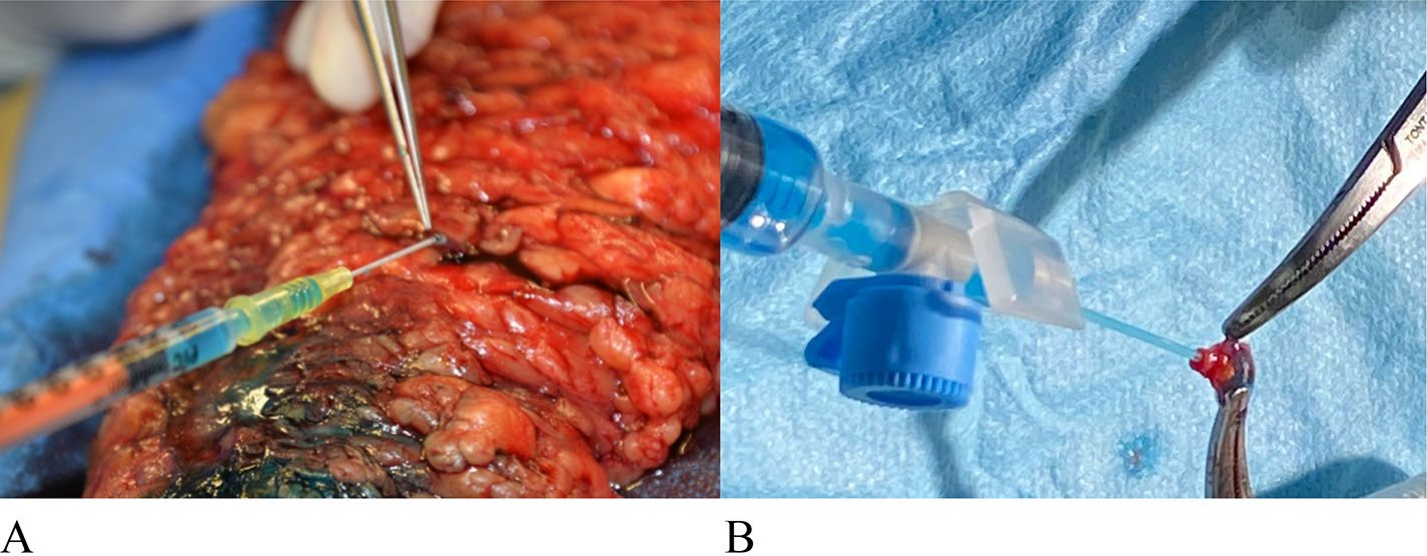
Fixative flush infused into the lymphatic vessel (**A**) and into the vein (**B**).

The excised tissue was dissected outside the operating table under Loupes 3× magnification by means of microsurgical instruments, searching for the patent blue-labelled subcutaneous lymphatic vessels. Once the lymphatic vessels were identified, their lumen was washed with a delicate flush of saline solution, followed by the dedicated fixative for electron microscopic analysis, the precise composition of which is reported in the section below. The largest needle suitable for the flush (30-gauge) was used, which indicated a vessel diameter of approximately 0.3 mm. After fixation, the lymphatic vessel was atraumatically removed and inserted into a vial filled with the same fixative. A vein adjacent to the harvested lymphatic was sent to the laboratory as well, following the same dissection and fixation technique (Fig. 1).

The study design was approved by the local Institutional Review Board (University of Padova, N. 2658P). The study was conducted according to the criteria set by the declaration of Helsinki. The subject signed the informed consent.

### Transmission electron microscopy

The specimens were divided into two fragments, one for histological analyses by light microscopy and the other for morphological analyses by transmission electron microscopy (TEM). For TEM analyses, the lymphatic vessels and veins were fixed in 2.5% glutaraldehyde in 0.1 M cacodylate buffer (pH 7.4), containing 0.05% (w/v) Alcian Blue 8GX, at 4 °C over-night and post-fixed in 2% buffered osmium tetroxide for 1 h. The specimens were then dehydrated with graded concentrations of acetone and embedded in Araldite epoxy resin (Durcupan ACM, Fluka, Sigma-Aldrich Co., St. Louis, MO, USA) according to standard protocols.

For orientation, semi-thin sections (1.5 μm) were cut on a Reichert Ultracut S ultramicrotome using glass knives and stained with a 1% aqueous solution of toluidine blue and examined with an optical microscope (Nikon Eclipse E800). Ultrathin sections (90 nm) were prepared with an ultramicrotome (Reichert UltracutS) and counterstained with uranyl acetate in saturated solution and lead citrate according to Reynolds and observed under transmission electron microscope (TEM Zeiss EM 910, Zeiss, Wetzlar, Germany) at 80,000× magnification. The depth of the glycocalyx was measured near the surface, where the plasma membrane was visible.

### Immunohistochemistry

For histological analyses the lymphatic and venous samples were fixed in formalin 10% for 24 h at 4 °C, dehydrated through an alcohol series and then paraffin-embedded using a Shandon Citadel 2000 Tissue Processor (Thermo Fisher Scientific, Waltham, MA). Five-μm-thick sections were cut from paraffin blocks. The sections were stained with the primary anti-podoplanin,, anti-glypican-1, anti-agrin, and anti-brevican antibodies, all from Abcam (Cambridge, UK), and the anti-mucin-2 antibody (Thermo Fisher Scientific), and then counterstained with the anti-rabbit HRP-DAB tissue staining kit (R&D Systems, Minneapolis, MN). A negative control was obtained in each slide by carrying out the immunohistochemistry staining procedure without using the primary antibody. The images were acquired with an Aperio ScanScope^®^ slide scanner by using the Aperio ImageScope v11.1.2.760 software (Leica Biosystems, Nussloch, Germany).

## Results

**Figure 2:**
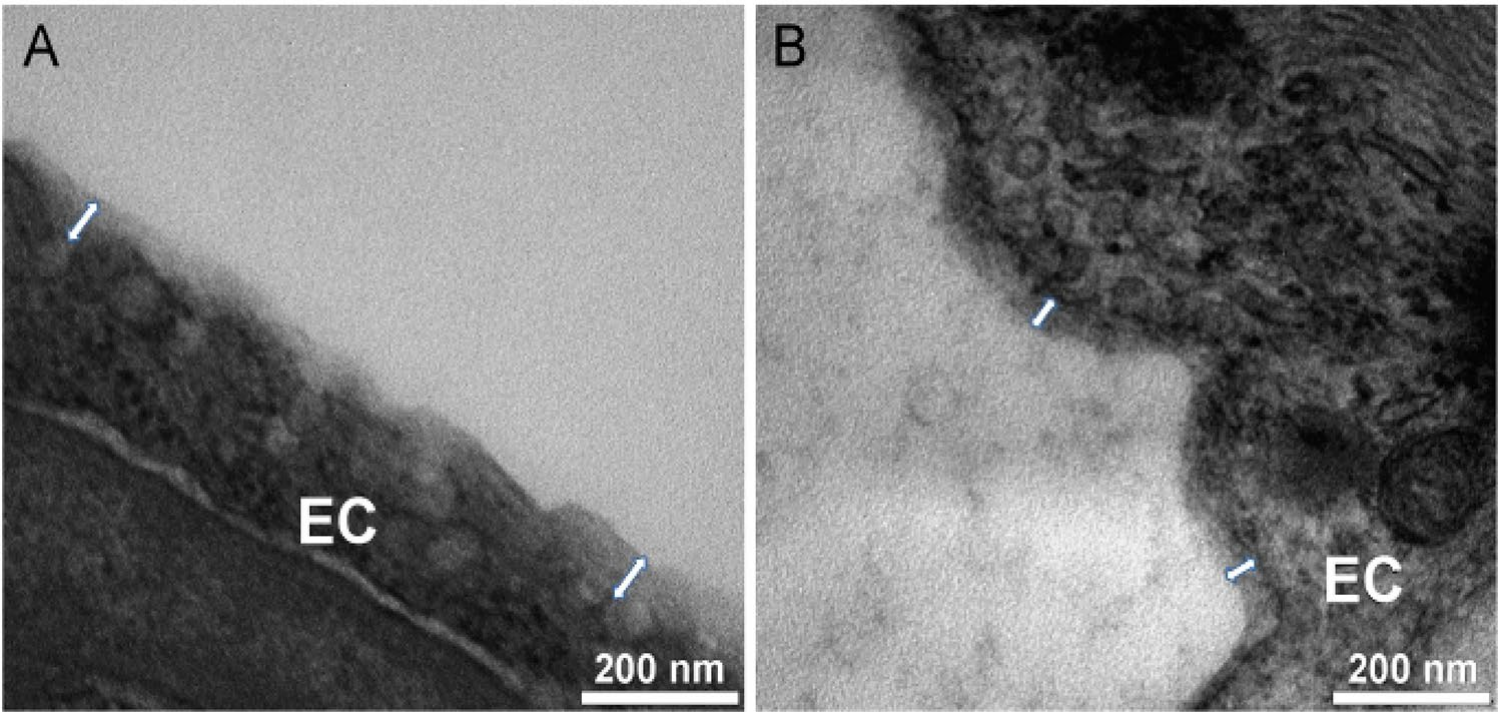
Glycocalyx-like structure assessment of lymphatic vessels. High-magnification transmission electron micrographs (scale bar: 200 nm) showing the endothelial glycocalyx-like structure in a vein (**A**) and in a post-valve lymphatic vessel (**B**) fixed with glutaraldehyde in the presence of Alcian Blue 8GX. EC indicates endothelial cells. Double white arrow indicates the glycocalyx layer.

After fixation, the wall thickness of the vein and of the lymphatic samples measured X10 along the vessel transverse section, were 397.3 ± 208.3 mm (min = 179 mm; max = 719.1 mm), and 51.3 ± 12.6 mm (min = 32.55 mm; max = 71.26 mm), respectively. TEM analysis confirmed the presence of a GCX-like structure along both the vein (Fig. 2A) and lymphatic (Fig. 2B) endothelium. The mean height of this structure measured 17X along the vein section and 25X along the lymphatic transverse section was 66.94 ± 18.38 nm (min = 53.4 nm; max = 113.09 nm) in the vein, and 49.26 ± 11.30 nm (min = 26.61 nm; max = 67.83 nm) in the lymphatic sample (p < 0.001). Table 1 summarizes the vein and lymphatic measurements.

**Table 1:** Mean, minimum and maximum values of vein and lymphatic wall (W) and glycocalyx-like structure (GCX) thickness. GCX/W indicates the glycocalyx-like/wall thickness ratio.

	Vein	Lymph
Mean GCX thickness (nm)	66.94 ± 18.38	49.26 ± 11.30
Min GCX thickness (nm)	53.4	26.61
Max GCX thickness (nm)	113.09	67.83
Mean wall (W) thickness (mm)	397.3 ± 208.3	51.3 ± 12.6
Min wall (W) thickness (mm)	179	32.55
Max wall (W) thickness (mm)	719.1	71.26
GCX/W mean	0.02%	0.1%
GCX/W min	0.03%	0.08%
GCX/W max	0.02%	0.1%

In both the vein and lymphatic samples, an amorphous structure was detected along the endothelium lining, featuring a lighter Alcian Blue staining when compared to the plasma membrane.

The vein sample was homogeneously covered by the amorphous structure along the entire length of the endothelial lining. The lymphatic amorphous layer showed interrupted zones along areas of endothelial lining disruption.

Immunohistochemical analyses was performed in various sections of the lymphatic vessel and vein by means of antibodies to anti-podoplanin, anti-glypican 1, and anti-mucin-2 (scale bar: 50 μm). Black arrows were added within the images to allow for better identification of the specific labelling.

**Figure 3:**
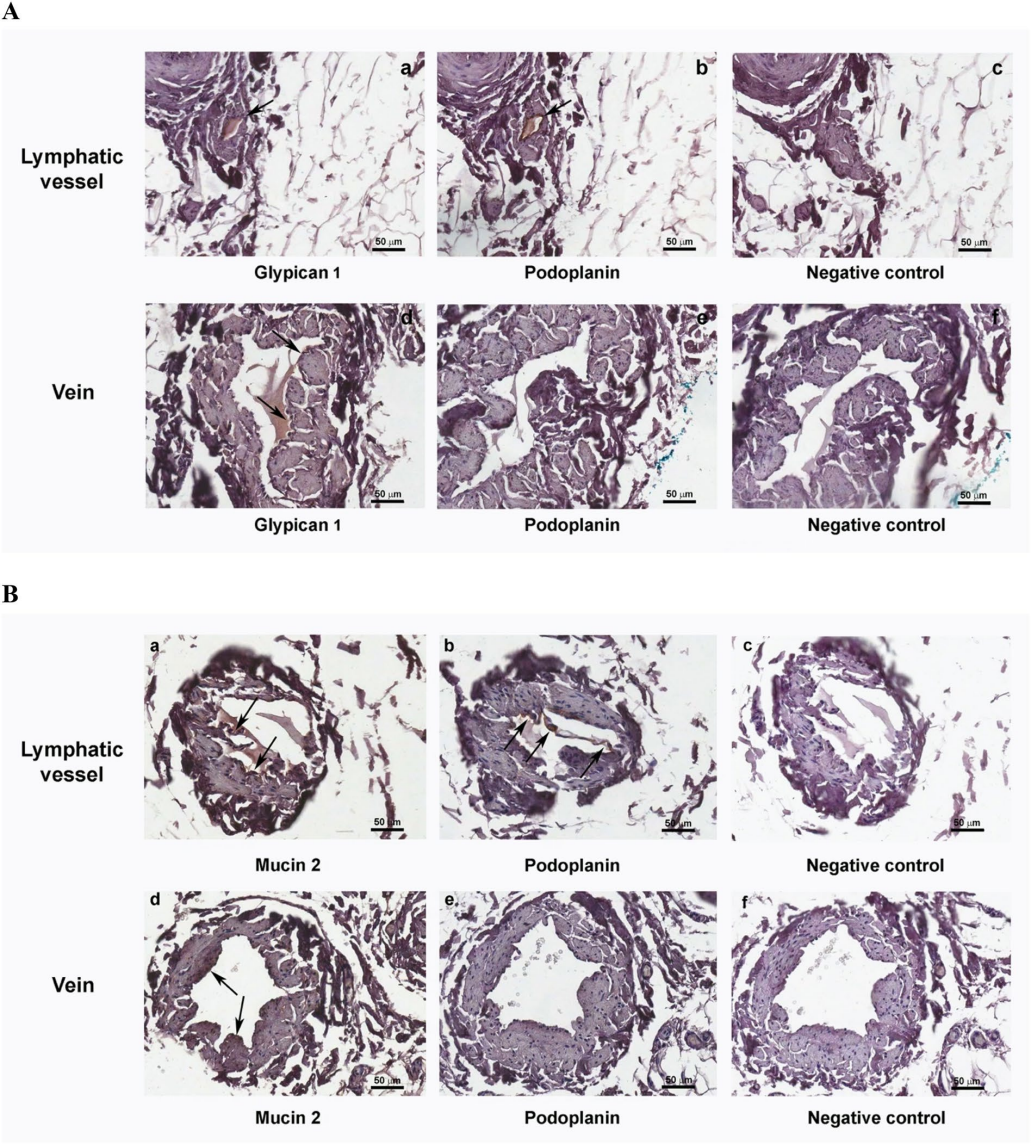
Immunohistochemistry showing lymphatic glycocalyx-like structure components.

Both venous and lymphatic immunohistochemistry identified glypican-1 and mucin-2. Agrin and brevican were identified in both the vein and lymphatic samples, and on all of the external surfaces of vessel wall cells, interpreted to represent a diffuse component of the extracellular matrix. Therefore, both agrin and brevican were not considered as characterizing elements of the glycocalyx. All the results were confirmed by the negative control performed by the secondary antibody (Fig. 3). Podoplanin, widely recognized as a lymphatic tissue-specific marker, was identified only in the lymphatic vessel (Fig. 3B)^33^.

## Discussion

Veins and lymphatics are strictly interconnected and mutually involved in lower limb fluid drainage^34^. This observation, together with the shared embryological origin of venous and lymphatic endothelium, led us to explore the suspected presence of a lymphatic GCX in human tissue^35^. While investigations of animal models have previously characterized moieties derived from lymphatic tissue^32^, the literature is lacking in ex vivo morphological and immunohistochemical characterizations of human samples, especially comparatively with venous tissue derived from the same human subject. To our knowledge, the findings presented here are the first in human ex vivo identification of a lymphatic glycocalyx-like structure. The characterization of a vein sample from the same patient also offers a unique opportunity to compare the GCX-like ultrastructure and composition originating, respectively, from lymphatic and venous tissues. The TEM clearly showed an amorphous structure along the endothelial surface of both vein and lymphatic samples. The mean thickness of the venous GCX-like structure was greater than in the lymphatic, but the ratio between the GCX-like structure and the wall thickness was greater in the lymphatic segment (Table 1).

These observations correlate with previous reports of varying GCX morphology within different anatomical locations, and may be an inherent requirement of the lymphatic endothelial cells in regulating fluid and macromolecule transport from the interstitial space and for immunomodulation^31,36,37^. We hypothesize that a low fluid force is gently acting on the endothelium, allowing the growth of a thicker GCX compared to its counterparts covering the endothelial linings exposed to higher hemodynamic forces, where a disruptive mechanical action might operate on the delicate surface structures. Indeed, in vein and arteries, GCX thickness has been reported to vary between 20 and 400 nm in veins and approximately 500 nm in arteries on average, reaching up to 4.5 μm in the carotid artery^38,39^.

The low flow in the lymphatics, when compared to the more turbulent venous flow, could explain our current observations when we consider the ratio between the thickness of the GCX and that of the vascular wall. Nevertheless, this hypothesis is not sustainable if the absolute thickness values are considered. Further investigations are needed to explore this hypothesis, and would require vein and lymphatic samples of equivalent anatomical and hemodynamic features to understand the anatomical and functional relationships.

No difference between the lymphatic and venous density of the GCX-like structure were demonstrated in the TEM of the patient studied, and a validated measurement method is currently lacking; therefore, statistical analysis of this morphologic aspect is not feasible here. Considering the intricate mesh-like structure of the GCX and its related functionality, direct observation of its morphological and immunohistochemical features is of paramount importance. Visualization techniques using an enzymatic wash to detect the different GCX components is essential for understanding the components involved in the structure. Nevertheless, the complex, three-dimensional architecture of the GCX requires ex vivo direct visualization of its entirety in order to derive proper insights.

The challenges surrounding sample fixation for microscopic analysis are well known. The GCX is an extremely fragile structure, easily altered, injured, and dehydrated during vessel handling and preparation. This has been evident already in investigations of the more robust arterial and venous structures and becomes particularly challenging in the current observations of the delicate lymphatic endothelium. Such limitations should be taken into consideration whenever reporting the GCX dimensions, since GCX measurement following fixation could be significantly underestimated due to the loss of thickness in the fixation process^40^.

Moreover, the herein-presented electron microscopic imaging is biased by the possible representation of oblique images of a plasma membrane from a section or an amorphous structure on top of an indistinct membrane. Additionally, Alcian Blue is expected to stain the GCX darker, rather than lighter, when compared to the plasma membrane, whereas our results showed the opposite. This suggests the need to use the herein-reported fixation technique as a starting point for improving future sampling preparation along with the pioneering challenges that are present in vein and lymphatic scanning in humans rather than in animals. In order to confirm the presence of a GCX-like structure in the harvested venous and lymphatic tissues, and apart from the TEM observations outlined in this investigation, our research laboratory performed immunohistochemical analysis to characterize glypican-1 and mucin-2 in the vein and lymphatic GCX. As expected, podoplanin was detected only in the lymphatic sample, as it is a specific lymphatic tissue marker. We rely upon anti-podoplanin staining to distinguish the lymphatic vasculature from venous and arterial vessels within the anatomic specimens^33,41,42^.

Glypican-1 was identified in the vein and lymphatic GCX, correlating with previous reports regarding its function in endothelial homeostasis and its anti-atherosclerosis properties^43,44^. A recent review highlighted the role of glypican-1 in regulating multiple cellular signaling pathways, including fibroblast growth factors, vascular endothelial growth factor-A, transforming growth factor-β, and bone morphogenic protein^45^. We identified mucin-2 as another component of the lymphatic GCX. The presence of mucin within the GCX has been described for decades^46^. Interestingly, mucin-2 has more recently been demonstrated to occupy a role in preventing intercellular junction defects, which is a feature related to the known lymphatic functions in extracellular fluid homeostasis^47^.

We also detected brevican as another component. It is a proteoglycan previously identified in the nervous system, where it represents a key element of the peri-synaptic extracellular matrix. Its role as regulator of synaptic plasticity and creation of perineural nets creation suggests that further work is necessary to define its role in the function of the lymphatic matrix^48^.

Our immunohistochemical analysis also identified agrin in the lymphatic GCX. This is another proteoglycan recently investigated in the realm of angiogenesis regulation. Detection of agrin in the lymphatic GCX mandates further exploration of its role in lymphatic vessel development and proliferation^49,50^. However, it must be re-emphasized that neither brevican or agrin was found exclusively in the glycocalyx, rather, they also represent components of the extracellular matrix. Further investigation is needed to explore their potential role in mechanotransduction.

The findings of this study are compatible with a potential role of the GCX in lymphatic function and in the related fluid filtration. However, it must be stated that a glycocalyx layer is present on many other types of cells (e.g., fibrocytes, leukocytes, enterocytes, neurons, osteocytes, and endothelial cells), and thus its presence on lymphatic endothelium does not necessarily indicate that it performs the same functions (e.g., mechanotransduction, permeability control, coagulation balance, vessel contractility regulation) as the glycocalyx of the arterial and venous endothelium. Taking into consideration the previous investigations on the topic^51–54^, future research should prospectively assess a defined role for the lymphatic glycocalyx in modulation of permeability and immunomodulation balance.

This is a pilot study that stimulates further detailed characterization of what represents the first in human identification and visualization of the lymphatic GCX. The limitations of the present research include the single human subject nature of the analysis, the possible shedding of GCX components during harvesting and during the fixation flush, and the limited number of glycoproteins, proteoglycans, and glycosaminoglycans identified. The need for an advanced assessment methodology to subserve in vivo GCX investigation has been recently published by Haymet et al., addressing the need for static as well as dynamic models^50^.

Moreover, our data analysis was focused on collecting lymphatics in order to define the presence of the lymphatic glycocalyx and to provide initial chemical structure analysis. Further assessment will be needed to evaluate the different lymphatic vascular subtypes (pre-valve, valve and post valve zones) and locations in the human body, inasmuch as varying GCX structures might be identified in future analysis^55^. The translational importance of this bench investigation can be inferred from the intricate pathophysiology of diseases related to arterial, venous, and lymphatic mechanisms. A GCX assessment has been recently introduced into clinical practice through a dedicated sublingual test named “GlycoCheck”, with the ability to report GCX thickness^56^. Particularly in the COVID era, the role of endothelial inflammation and the importance of its ability to reverse harmful cardiopulmonary effects has been made clear to the scientific community. The GCX has already been identified as a potential treatment target for microvascular endotheliopathy^57^, and may be of considerable interest in the study and pathology of the lymphatic system.

## Conclusions

The present investigation provides the first in human identification and characterization of a GCX structure also present in the lymphatic system, providing a comparative analysis of ex vivo samples of lymphatic and venous tissue obtained from the same subject. The study provides fundamental information and technical applications for further assessments in the different parts of the lymphatic systems that may be both physiological and pathological conditions, and potentially providing translational resources that will inform the future management of the extremely relevant clinical issue of lymphedema.

## Data Availability

All data are fully available without restriction.
